# Special Issues of “Materials for Luminescent Detectors and Transformers of Ionizing Radiation”

**DOI:** 10.3390/ma16093319

**Published:** 2023-04-23

**Authors:** Yuriy Zorenko, Kazimierz Fabisiak, Janusz Winiecki

**Affiliations:** 1 Department of Physics, Kazimierz Wielki University, Powstańców Wielkopolskich Str., 2, 85-090 Bydgoszcz, Poland; 2 Medical Physics Department, Oncology Center in Bydgoszcz, Prof. Franciszek Łukaszczyk Memorial, dr I. Romanowska Str. 2, 85-796 Bydgoszcz, Poland; janusz@co.bydgoszcz.pl; 3 University of Bydgoszcz, Unii Lubelskiej Str. 4C, 85-059 Bydgoszcz, Poland; 4 Collegium Medicum in Bydgoszcz, Nicholas Copernicus University, Gagarina Str. 5/7, 87-100 Toruń, Poland

The papers published in the first and second Special Issues of “Materials for Luminescent Detectors and Transformers of Ionizing Radiation” were selected from the manuscripts related to the respective presentations at the 11th International Conference on Luminescent Detectors and Transformers of Ionizing Radiation (LUMDETR 2021), which was organized by the Institute of Physics of Kazimierz Wielki University of Bydgoszcz and Oncology Center prof. Franciszek Łukaszczyk in Bydgoszcz, from 12 to 17 September 2021.

LUMDETR 2021 will continue the tradition established by previous meetings in Latvia (Riga, 1991), Estonia (Tallin, 1994), Poland (Ustroń, 1997), Latvia (Riga, 2000), Czech Republic (Prague, 2003), Ukraine (Lviv, 2006), Poland (Krakow, 2009), Germany (Halle, 2012), Estonia (Tartu, 2015), and the Czech Republic (Prague, 2018).

It is worth noting that LUMDETR 2021 was the first conference in the post-coronavirus era organized in a mixt mode (on-site and on-line). The conference was attended by 144 participants from 23 countries, and most of them (97 participants) travelled to Bydgoszcz (Figure 1). 

The program of the LUMDETR 2021 conference consisted of 16 thematic oral sessions and 2 poster sessions. In total, 149 abstracts were accepted for presentation. This number included 32 keynote presentations, 65 oral presentations, and 52 poster presentations. We would like to extend our appreciation to the excellent scientific level of all presentations. 

The conference was preceded by the LUMDETR 2021 Summer School, organized from 10 to 12 September 2021 by the Institute of Physics of Kazimierz Wielki University in Bydgoszcz. The Summer School collected 22 PhD students from 6 different countries. (Figure 2) The 12 lectures, associated with the scintillators and detectors, as well as the advanced methods of their investigations, were delivered by 12 professors from Czech, Italy, Estonia, France, Ukraine, and Poland (Figure 2). We would also like to thank the Polish Ministry of Science and Higher Education for its support in the framework of “Excellent Science” Program for their financial support of the LUMDETR 2021 Summer School Organization.

We would also like to express our thanks to all conference participants due to their intendance in Bydgoszcz, as well as their contribution to the scientific program of the conference. We hope that the informal atmosphere of the meeting and the social program, including excursions to the historical part of Toruń and Golub-Dobrzyń castle, as well as the Gala dinner in Lubostroń Palace, will help to inspire further formal and unformal collaboration between participants of the conference in the future. 

We gratefully acknowledge the efficient work of all the members of the Local Organizing Committee in Bydgoszcz and to express our gratitude to members of the International Scientific Committee for their important contributions and guidance.

The Special Issues of “Materials for Luminescent Detectors and Transformers of Ionizing Radiation” contain selected high-level publications related to the latest developments in basic and applied research in the field of radioluminescence [1,2,3,4,5] and the processes of energy transfer and storage in solids [6,7,8,9,10,11,12]. The main aspect of the Special Issue is related to the physics, chemistry, and technology of scintillation and dosimetric materials based on single crystalline films, single crystalline filmsd and composite film-crystal epitaxial structures grown using liquid-phase epitaxy method [1,2,3,4,5,6,7]. Special attention in this Special Issue was also given to the development of new thermo- [3,4] and optically- simulated luminescent detectors [6,7], as well as related applications of them in medicine [8,9]. The last part of collected papers was related to the development of luminescent converters for white LED produced in different crystalline forms, including micropowders, films, film–crystal epitaxial structures, and eutectic compositions [10,11,12]. 

We also wish to extend our thanks to Ms Nancy Shao, M.Sc., Section Managing Editor of *Materials*, as well as all *Materials* staff for providing us with the possibility of publishing selected papers from the LUMDETR 2021 conference in the first and second Special Issues of “Materials for Luminescent Detectors and Transformers of Ionizing Radiation”. We would like to express our thanks to the authors and reviewers for their contribution to these Special Issues.

## Figures and Tables

**Figure 1:**
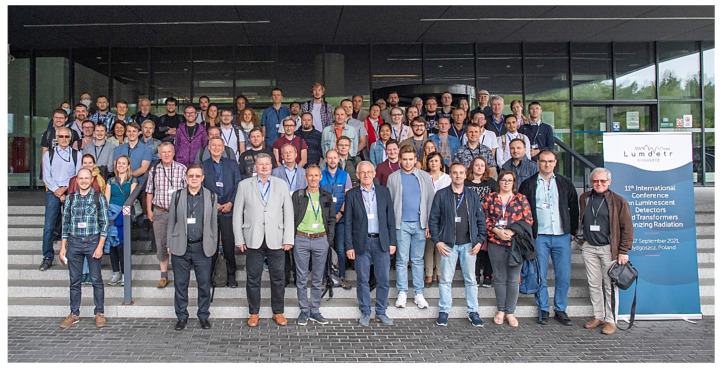
Photo of the LUMDETR 2021 conference.

**Figure 2:**
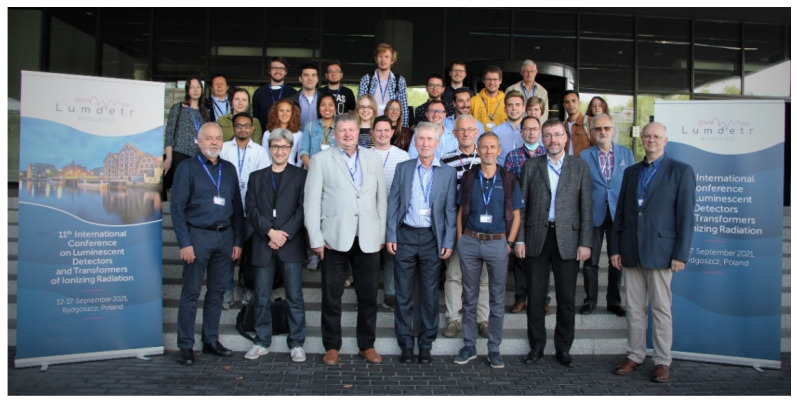
Photo of the LUMDETR 2021 Summer School.

## References

[1] Witkiewicz-Lukaszek S., Gorbenko V., Zorenko T., Syrotych Y., Mares J.A., Nikl M., Sidletskiy O., Bilski P., Yoshikawa A., Zorenko Y. (2022). Composite detectors based on single crystalline films and single crystals of garnet compounds. Materials.

[2] Mares J.A., Gorbenko V., Kucerkova R., Prusa P., Beitlerova A., Zorenko T., Pokorny M., Witkiewicz-Łukaszek S., Syrotych Y., D’Ambrosio C. (2022). Scintillating Characteristics of the Single-Crystalline Film and Composite Film-Crystal Scintillators Based on the Ce^3+^-Doped (Lu,Gd)_3_(Ga,Al)_5_O_12_ Mixed Garnets under Alpha and Beta Particles, and Gamma Ray Excitations. Materials.

[3] Mrozik A., Bilski P., Gieszczyk W., Kłosowski M., Witkiewicz-Łukaszek S., Gorbenko V., Zorenko T., Zorenko Y. (2022). Application of the LPE-grown LuAG:Ce film/YAG crystal composite thermoluminescence detector for distinguishing the components of the mixed radiation field. Materials.

[4] Witkiewicz-Łukaszek S., Mrozik A., Gorbenko V., Zorenko T., Bilski P., Syrotych Y., Zorenko Y. (2022). Development of the composite thermoluminescent detectors based on the single crystalline films and crystals of perovskite compounds. Materials.

[5] Gorbenko V., Zorenko T., Shakhno A., Popielarski P., Osvet A., Batentschuk M., Fedorov A., Mahlik S., Leśniewski T., Majewska N. (2023). Single Crystalline Films of Ce^3+^-Doped Y_3_Mg_x_Si_y_Al_5−x−y_O_12_ Garnets: Crystallization, Optical, and Photocurrent Properties. Materials.

[6] Bilski P., Mrozik A., Gieszczyk W., Nizhankovsky S., Zorenko Y. (2022). Infrared stimulated luminescence of Ce^3+^ doped YAG crystals. Materials.

[7] Sankowska M., Bilski P., Marczewska B., Zhydachevskyy Y. (2023). Influence of Elevated Temperature on Color Centers in LiF Crystals and Their Photoluminescence. Materials.

[8] Winiecki J., Witkiewicz-Lukaszek S., Michalska P., Jakubowski S., Nizhankovskiy S., Zorenko Y. (2022). Basic characteristics of dose distributions of photons beam for radiotherapeutic applications using YAG:Ce crystal detectors. Materials.

[9] Sądel M., Grzanka L., Swakoń J., Baran J., Gajewski J., Bilski P. (2023). Optically Stimulated Luminescent Response of the LiMgPO_4_ Silicone Foils to Protons and Its Dependence on Proton Energy. Materials.

[10] Shakhno A., Markovskyi A., Zorenko T., Witkiewicz-Łukaszek S., Vlasyuk Y., Osvet A., Elia J., Brabec C.J., Batentschuk M., Zorenko Y. (2022). Micropowder Ca_2_YMgScSi_3_O_12_:Ce Silicate Garnet as Efficient Light Converter for White LEDs. Materials.

[11] Markovskyi A., Gorbenko V., Zorenko T., Witkiewicz-Lukaszek S., Sidletskiy O., Fedorov A., Zorenko Y. (2023). Development of Three-Layered Composite Color Converters for White LEDs Based on the Epitaxial Structures of YAG:Ce, TbAG:Ce and LuAG:Ce Garnets. Materials.

[12] Shakhno A., Zorenko T., Witkiewicz-Lukaszek S., Cieszko M., Szczepański Z., Vovk O., Nizhankovskiy S., Siryk Y., Zorenko Y. (2023). Ce^3+^ doped Al_2_O_3_-YAG eutectic as an efficient light converter for white LEDs. Materials.

